# Chronic Skull Base Erosion from Temporomandibular Joint Disease Causes Generalized Seizure and Profound Lactic Acidosis

**DOI:** 10.1155/2018/8795036

**Published:** 2018-09-27

**Authors:** Mark A. Dobish, David A. Wyler, Christopher J. Farrell, Hermandeep S. Dhami, Victor M. Romo, Daniel D. Choi, Travis Reed, Michael E. Mahla

**Affiliations:** ^1^Anesthesiology Resident, Department of Anesthesiology, Thomas Jefferson University Hospital, Philadelphia, PA, USA; ^2^Assistant Professor of Anesthesiology and Critical Care Medicine, Department of Anesthesiology, Thomas Jefferson University Hospital, Philadelphia, PA, USA; ^3^Assistant Professor of Neurological Surgery, Department of Neurosurgery, Thomas Jefferson University Hospital, Philadelphia, PA, USA; ^4^Medical Student, Sidney Kimmel Medical College, Thomas Jefferson University Hospital, Philadelphia, PA, USA; ^5^Assistant Professor of Anesthesiology, Department of Anesthesiology, Thomas Jefferson University Hospital, Philadelphia, PA, USA; ^6^Assistant Professor of Oral and Maxillofacial Surgery, Department of Oral and Maxillofacial Surgery, Thomas Jefferson University Hospital, Philadelphia, PA, USA; ^7^Resident, Department of Oral and Maxillofacial Surgery, Thomas Jefferson University Hospital, Philadelphia, PA, USA; ^8^Professor and Executive Vice Chair of Anesthesiology, Department of Anesthesiology, Thomas Jefferson University Hospital, Philadelphia, PA, USA

## Abstract

This report displays a rare presentation of lactic acidosis in the setting of status epilepticus (SE). The differential diagnosis of lactic acidosis is broad and typically originates from states of shock; however, this report highlights an alternative and rare etiology, SE, due to chronic skull base erosion from temporomandibular joint (TMJ) disease. Lactic acidosis is defined by a pH below 7.35 in the setting of lactate values greater than 5 mmol/L. Two broad classifications of lactic acidosis exist: a type A lactic acidosis which stems from global or localized tissue hypoxia or a type B lactic acidosis which occurs once mitochondrial oxidative capacity is unable to match glucose metabolism. SE is an example of a type A lactic acidosis in which oxygen delivery is unable to meet increased cellular energy requirements. This report is consistent with a prior case series that consists of five patients experiencing generalized tonic-clonic (GTC) seizures and lactic acidosis. These patients presented with a pH range of 6.8-7.41 and lactate range of 3.8-22.4 mmol/L. Although severe lactic acidosis following GTC has been described, this is the first report in the literature of chronic skull base erosion from TMJ disease causing SE.

## 1. Introduction

Status epilepticus (SE) is a neurological emergency with a reported mortality rate between 7.6% and 43% [1]. The three most common etiologies of SE in adults are anticonvulsant noncompliance, alcohol withdrawal, and central nervous system infections which have a reported incidence of 29%, 26%, and 8%, respectively [2]. Other less common etiologies of seizures and SE are rare autoimmune and paraneoplastic disorders, hereditary mitochondrial disease, and rare chromosomal disorders [3]. Trauma is a common source of SE and is attributed as the causative factor in approximately 8% of cases [4].

Lactic acidosis commonly accompanies status epilepticus and is recognized as a transient phenomenon. This is due to over-activity of both neurons and skeletal muscle leading to anaerobic metabolism and the production of lactic acid. Concentrations of lactic acid have been shown to decrease rapidly following SE [5], such that other etiologies should be investigated if this does not occur. Other common sources of lactic acidosis include shock, sepsis, occult malignancy, liver disease, genetic conditions, and medication side effects.

Displacement of the condylar head into the middle cranial fossa is an extremely rare event. A literature review reveals 55 cases of displaced condyles into the middle cranial fossa, all the result of trauma [6, 7]. We describe a case report of chronic temporomandibular joint (TMJ) osteoarthritis causing severe erosive changes in the glenoid fossa leading to intracranial edema, new onset SE, and intracranial displacement of the mandibular condyle.

## 2. Case Report

A 65-year-old patient developed acute onset aphasia at her home followed by SE while conversing with her sister on the phone. Seizure activity was witnessed by paramedics upon arrival. Due to the inability to open the patient's oral cavity, a blind nasal intubation was performed at the scene.

**Figure 1:**
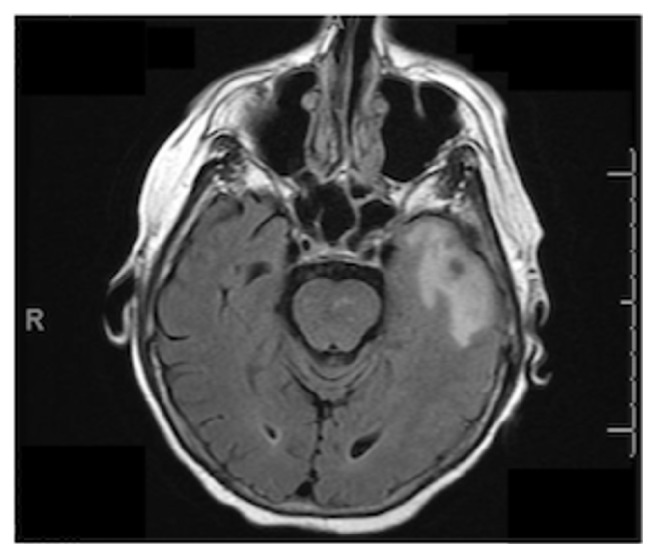
Axial flair MRI showing vasogenic edema of the temporal lobe.

**Figure 2:**
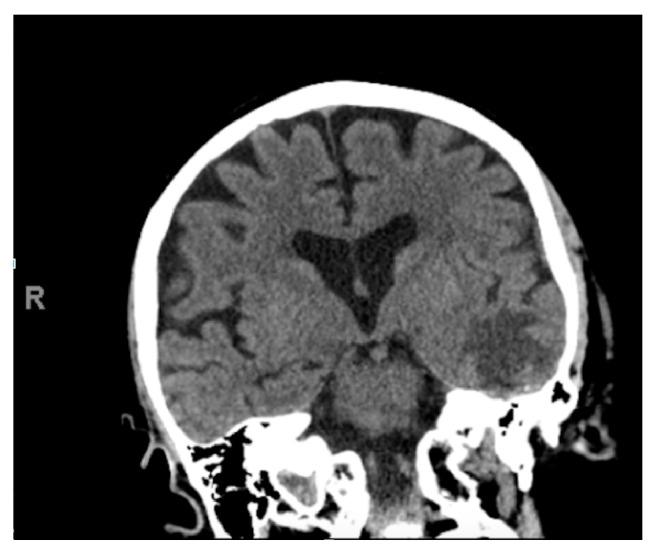
Coronal CT demonstrates hypodense left temporal lobe vasogenic edema.

**Figure 3:**
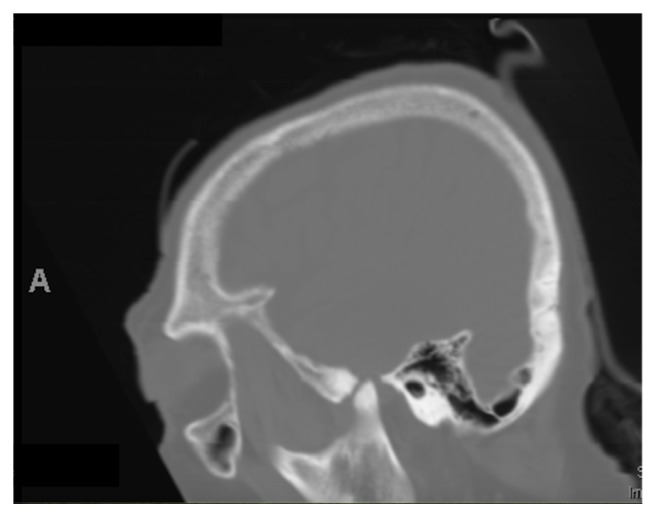
Sagittal CT demonstrates erosion of the skull base and protrusion of the TMJ.

**Figure 4:**
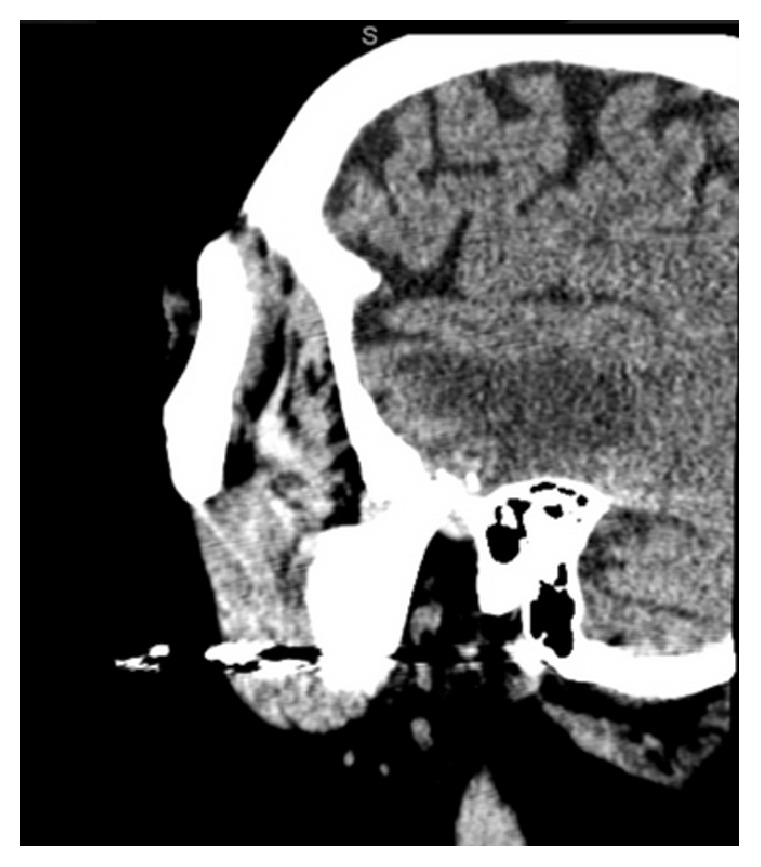
Soft tissue window sagittal CT illustrating intracranial edema surrounding the glenoid fossa.

Upon arrival to the emergency department, SE was treated with bolus doses of midazolam followed by a levetiracetam load. A propofol infusion was started for sedation. Initial laboratory findings were significant for a metabolic acidosis with pH 6.9, CO_2_ 80 mEq/L, and lactate 11.0 mmol/L. Computed tomography (CT) imaging showed vasogenic edema in the left temporal lobe secondary to intracranial displacement of the left mandibular condyle through a 1.3 × 0.7 cm defect of the glenoid fossa of the temporal bone. Advanced osteoarthritic changes were noted in bilateral TMJ spaces as well as asymptomatic thinning and perforation of the contralateral glenoid fossa. MRI confirmed these findings and further portrayed abnormally enhancing soft tissue that surrounded the mandibular condyle that likely represented fragmented pieces of the glenoid fossa (Figures 1–4).

Three days following admission, the patient was extubated without complication and plans were arranged for surgical repositioning of the mandibular condyle and skull base reconstruction. The patient reported a history of chronic TMJ osteoarthrosis for more than twenty years with a progressive shift in occlusion over the past several years. The patient denied any previous history of seizures, traumatic incidents or TMJ surgical interventions. On exam, there was significant trismus, a mildly canted mandible, and over-collapsed left posterior dentition consistent with an intracranially displaced mandibular condyle.

Based on this preoperative assessment, a difficult airway was anticipated. Furthermore, all jaw manipulation was avoided due to the temporal glenoid defect. An asleep nasal intubation was planned and the patient was brought to the operating room. Standard monitors were applied and each nare was premedicated with two sprays of oxymetazoline in the sitting position. Following pre-oxygenation, general anesthesia was induced with fentanyl, lidocaine, propofol, and rocuronium. The right nare was then dilated using 28, 30, and 32 French lubricated nasal airways. A 6.5mm nasal endotracheal tube (ETT) was advanced atraumatically into the posterior oropharynx and ventilation at this position was confirmed prior to further airway instrumentation. Next, a McGrath 3 blade was carefully inserted into the vallecula and a Cormack-Lehane Grade I view of the laryngeal inlet was obtained with minimal jaw manipulation. The ETT passed through the cords atraumatically. Following positioning and mayfield pinning, a portable bronchoscope was used to confirm final ETT location and the tube was secured at 26 cm at the right nare.

The patient's intraoperative course lasted approximately eleven hours during which time the patient underwent a left middle fossa craniotomy, TMJ arthroplasty, glenoid fossa reconstruction with split-thickness calvarial bone graft, and maxillomandibular fixation. The surgery was performed by a team of neurosurgeons and oral and maxillofacial surgeons. Prior to incision, the patient received mannitol 1 g/kg, dexamethasone 10 mg, and furosemide 20 mg to reduce cerebral edema and optimize surgical conditions. Urine output was replaced in a 1:1 fashion with 0.9% normal saline. Maintenance of anesthesia was accomplished with a total intravenous technique consisting of propofol, remifentanil, and phenylephrine to support blood pressure as needed. At the conclusion of the procedure, the patient's neuromuscular blockade was fully reversed. She was awakened for a “neurologic wake-up test” which was normal. This was accomplished on dexmedetomidine 0.5 mcg/kg/hr and remifentanil 0.05 mcg/kg/min. The patient remained intubated due to a concern for glottic swelling and was taken to the ICU where she was extubated two days later. The patient was seizure free for the remainder of the hospitalization and was discharged on postoperative day five. At 6 months follow up, the patient continues to do well with resolution of aphasia, no residual weaknesses, and no further episodes of seizures.

## 3. Discussion

To our knowledge, this is the first case that presents SE caused by skull base erosion of the TMJ. The presumed etiology, supported by imaging, is an arthropathy that slowly weakened the TMJ causing progressive intracranial swelling and edema deep to the glenoid fossa. Intraoperative pathology indicated non-inflammatory degenerative joint pathology without cellular infiltrates, indicating a diagnosis of TMJ osteoarthritis. This ultimately caused SE, and the motor involvement caused the mandibular condyle to protrude intracranially through the perforated glenoid fossa.

Displacement of the mandibular condyle into the middle cranial fossa is an extremely rare finding due to the unique and protective anatomy of the TMJ complex. The glenoid fossa, although often as thin as 1 mm, rarely receives a large impactful force in its central portion due to the rounded nature of the condylar head. Additionally, any traumatic force distributed through the mandible has a tendency to fracture at the condylar neck as a protective measure. The patient was found sitting in a chair by EMS; therefore a fall or mechanical trauma was unlikely to have accounted for this finding. Additionally, an asymptomatic perforation of the contralateral glenoid fossa and progressive shift in occlusion support the chronic nature TMJ osteoarthritis.

The severity of this seizure is further illustrated by the lactic acidosis found on arrival - pH 6.9 and lactate 11.0 mmol/L. Since EMS responded immediately, these values are unlikely to have resulted from prolonged muscle breakdown. Initial creatinine phosphokinase value was normal, 166 U/L.

The differential diagnosis of lactic acidosis is broad and can occur as a result of genetic conditions, medication side effects, malignancy, or pathologic conditions that cause sepsis, anemia, liver dysfunction, or decreased cardiac output. Basal lactate production is about 0.8 mmol/kg/hour [8], and a normal reference range is typically 2 mmol/L or less [9]. Lactic acidosis is defined by a pH below 7.35 in the setting of lactate values greater than 5 mmol/L [10]. Lactate levels are routinely used as a marker for tissue hypoperfusion, and in cases of septic shock values greater than 4 mmol/L have been used as a threshold for aggressive fluid therapy [11]. Two broad classifications of lactic acidosis exist: a type A lactic acidosis which stems from global or localized tissue hypoxia or a type B lactic acidosis which occurs once mitochondrial oxidative capacity is unable to match glucose metabolism [12]. Causes of a type B lactic acidosis occur due to mitochondrial dysfunction and include, but are not limited to, malignancy, trauma, medication side effects, thiamine deficiency, or mitochondrial myopathy [13]. SE is an example of a type A lactic acidosis in which oxygen delivery is unable to meet increased cellular energy requirements, leading to anaerobic metabolism. In this state of localized hypoxia in the brain, pyruvate from cellular glycolysis is converted into lactate in the liver where it is metabolized back to glucose by the Cori cycle.

This case is consistent with two prior case series. The first case series consists of five patients experiencing SE in Scandinavia - these patients presented with a pH range of 6.8-7.41 and lactate range of 3.8-22.4 mmol/L [14]. The second case series of 8 patients, published in 1977, found an average pH of 7.14 (6.86-7.36mmol/L) and an average lactate of 6.6 mmol/L (4.2-10.7 mmol/L) [15].

Treatment options of intracranial condylar dislocation consist of closed reduction and open reduction, with or without reconstruction of the glenoid fossa. Decision making depends on timing of presentation and presence of neurologic symptoms. In the acute setting, an attempt should be made to manually reduce the displaced condyle followed by a period of MMF. However, when there is delayed presentation, it often necessitates an open approach to free the fibrosed mandibular condyle. Fossa reconstruction options described in the literature include autogenous grafts (cartilage or bone), titanium mesh, and alloplastic materials [16–18].

In conclusion, the most common causes of SE are anticonvulsant noncompliance, alcohol withdrawal, and nervous system infections. While seizures can result from traumatic intracranial dislocation of the mandibular condyle, this is the first known report describing long-standing TMJ disease leading to intracranial swelling and edema followed by new onset SE.
